# Biosynthesis and Characterization of Silver Nanoparticles Using *Tribulus terrestris* Seeds: Revealed Promising Antidiabetic Potentials

**DOI:** 10.3390/molecules28104203

**Published:** 2023-05-20

**Authors:** Abdur Rahman, Gauhar Rehman, Nasrullah Shah, Muhammad Hamayun, Sajid Ali, Abid Ali, Said karim Shah, Waliullah Khan, Muhammad Ishaq Ali Shah, Abdulwahed Fahad Alrefaei

**Affiliations:** 1 Department of Zoology, Abdul Wali Khan University Mardan, Mardan 23200, Pakistan; 2 Department of Chemistry, Abdul Wali Khan University Mardan, Mardan 23200, Pakistan; 3 Department of Botany, Abdul Wali Khan University Mardan, Mardan 23200, Pakistan; 4 Department of Horticulture and Life Science, Yeungnam University, Gyeongsan 38541, Republic of Korea; 5 Department of Physics, Abdul Wali Khan University Mardan, Mardan 23200, Pakistan; 6 Department of Zoology, College of Science, King Saud University, Riyadh P.O. Box 2455, Saudi Arabia

**Keywords:** anti-diabetic, green synthesis, silver nano-particles, *Tribulus terrestris*

## Abstract

Green synthesis is the most effective and environmentally friendly way to produce nanoparticles. The present research aimed at the biosynthesizing of silver nanoparticles (AgNPs) using *Tribulus terrestris* seed extract as the reducing and stabilizing agent and investigating their anti-diabetic properties. Fourier transformation infrared (FTIR), X-ray diffraction (XRD), scanning electron microscopy (SEM), and UV-Vis spectroscopy were used to analyze the synthesized silver nanoparticles from *Tribulus terrestris* (TT-AgNPs). The spectroscopic characterization revealed a surface Plasmon resonance band at 380 nm, which verified the development of TT-AgNPs. The transmittance peaks were observed at 596, 1450, 1631, 2856, 2921, and 3422 cm^−1^ through the FTIR spectrophotometer. The XRD spectrum showed four distinct diffraction peaks in the 2θ range at 20° to 60°. Intense peaks were at 26.32°, 30.70°, 44.70°, 56.07°, 53.75°, 66.28°, and 75.32°. The SEM analysis revealed that the prepared TT-AgNPs were clustered loosely with a smooth and spherical structure and were of relatively uniform size. The in vitro antidiabetic potential of TT-AgNPs was assessed by using glucose yeast uptake, glucose adsorption, and alpha-amylase assays. TT-AgNPs showed the highest activity (78.45 ± 0.84%) of glucose uptake by yeast at 80 µg/mL. In the glucose adsorption assay, the highest activity of TT-AgNPs was 10.40 ± 0.52% at 30 mM, while in the alpha-amylase assay, TT-AgNPs exhibited the maximum activity of 75.68 ± 0.11% at 100 µg/mL. The results indicate a substantial anti-diabetic effect of the TT-AgNPs. Furthermore, the in vivo antidiabetic study was performed on TT-AgNPs in streptozotocin-induced diabetic mice. After receiving TT-AgNPs treatment for 30 days, the mice were sacrificed for biochemical and histological analyses of pancreatic and liver samples, which demonstrated a good improvement when compared to the control group. Mice treated with TT-AgNPs showed a significant drop in blood sugar levels, showing that the biosynthesized TT-AgNPs have effective anti-diabetic properties.

## 1. Introduction

Nanotechnology has grown into a significant area of research in recent years, especially in the fields of health and medicine. Nanomedicine has shown a significant and far-reaching impact on healthcare and has opened numerous possibilities in various scientific disciplines and industries. Nanotechnology has gained great attention in the fields of drug encapsulation and targeted delivery. NPs are more compatible with the body than traditional therapeutics, allowing for increased drug efficacy and reduced toxicity. Silver nanoparticles (AgNPs) demonstrated distinctive properties, including high conductivity and chemical stability, with potential applications in antibacterial, antifungal, anticancer, antidiabetic, and anti-inflammatory activities [1]. Noble metal nanostructures have recently gained popularity and are used in a range of scientific and medical fields, including molecular imaging and medication delivery. [2], and the development of diagnostic and therapeutic materials and devices [3]. Metal nanoparticles can be produced using a variety of techniques, such as chemical reduction, microwave and UV radiation, photochemistry, and sonoelectrochemistry [4]. However, the use of toxic chemicals in these methods has led to a need for alternative, eco-friendly, and straightforward synthetic methods that utilize the reducing properties of natural compounds. The study of nanomaterials has been emerging dramatically throughout the world in the 21st century due to their incredible applications in all spheres of human life. It has opened several arms in the development of new nanomaterials and examining their properties by tuning the particle size, shape, and distribution [5]. Metal nanoparticles have been extensively studied due to their specific characteristics, such as catalytic activity, optical properties, electronic properties, antimicrobial, anti-inflammatory, anticancer, anti-diabetic, and magnetic properties [6]. Traditionally, UV irradiation, aerosol technologies, lithography, laser ablation, ultrasonic fields, and photochemical reduction techniques have been used successfully to produce various metal nanoparticles such as gold, silver, platinum, and palladium. The synthesis of novel metal nanoparticles, in particular silver nanoparticles (AgNPs), using natural organisms and plants has become a major research area in this field of nanotechnology. This may be due to their simplicity of procedures, stability of nanoparticles, and potential applications in chemical sensing, biological imaging, antimicrobials, gene silencing, and drug delivery [7]. Recently, several studies have reported natural polymers such as chitosan, starch, and tannic acid as reducing agents for the synthesis of silver and gold nanoparticles [8]. A vast array of biological resources, including plants, algae, fungi, yeast, bacteria, and viruses, have been studied so far for the intracellular and extracellular synthesis of silver, gold, platinum, and titanium nanoparticles in different sizes and shapes [9].

Silver nanoparticles made from plant or fruit extracts through phytochemical synthesis are a crucial aspect of nanomedicine and nanotechnology, as they provide safe, effective, and safe therapeutic options for a variety of diseases. Studies have shown that AgNPs have strong microorganism-inhibiting properties [10] and possess free radical scavenging and anti-inflammatory properties [11]. Additionally, AgNPs have been found to have wound healing properties, anti-tumor [12], antiviral, antibacterial, and anti-angiogenic effects [13]. AgNPs have been shown to trigger apoptosis and reduce the level of matrix metalloproteinase in wounds, limiting angiogenesis [14] and vascular permeability with the help of growth factors, i.e., VEGF, interleukin (IL)-1, and advanced glycation in endothelial cells of the retina [15]. However, the effect of AgNPs on diabetes is an area that has not been extensively studied [16].

Diabetes mellitus is one of the most frightening, widespread, and pandemic non-communicable chronic diseases in the world, which places a heavy burden on healthcare systems [17]. The quality of life in diabetic patients gets affected, and long-term secondary issues may damage the heart, kidneys, eyes, nerves, and blood vessels. Insulin is primarily responsible for glucose regulation; uncontrolled hepatic glucose production and hyperglycemia are metabolic diseases due to the abnormal insulin production in people with diabetes [18,19]. To control and cure DM, it requires changes in lifestyle, insulin, and oral hypoglycemic medications. Most of the drugs have severe side effects, are expensive, and are not readily available. Hence, a good proportion of the population uses traditional medicines in the care of DM, as plants are safer, more effective, and more affordable, making it possible to screen plants that naturally exhibit antidiabetic properties [20].

Pants can be used for the biosynthesis of nanoparticles because they are prominent sources of reducing agents. The biosynthesis of AgNPs from various plant sources, such *as Chrysanthemum morifolium*, *Cassia auriculata*, *Mimusops elengi*, *Cinnamon zeylanicum*, *Jatropha curcas*, and *Morinda citrifolia*, is illustrated by several examples [21]. Parts of plants such as leaves, seeds, roots, etc. are usually used for the biosynthesis of AgNPs. Plant constituents, i.e., carbohydrates, fats, enzymes, flavonoids, terpenoids, polyphenols, and alkaloids, can reduce silver to nanoparticles [21].

Because of their diverse physical and chemical properties, silver nanoparticles (AgNPs) are increasingly used in a wide range of industries, including those related to medicine, food, and health care, to name a few [22], as well as in drug delivery, such as anti-cancer drug delivery, and ultimately improving the effects of anti-cancer drugs [23]. More recently, existing AgNPs have been widely used in wound fabrics and biomedical tools [24]. In this research, green synthesized silver nanoparticles have been produced from the seed extract of *Tribulus terrestris*, and considering the therapeutic properties of *Tribulus terrestris* seeds, the anti-diabetic properties of these silver nanoparticles were evaluated.

## 2. Results

### 2.1. Synthesizing of Silver Nanoparticles (AgNPs)

**Figure 1:**
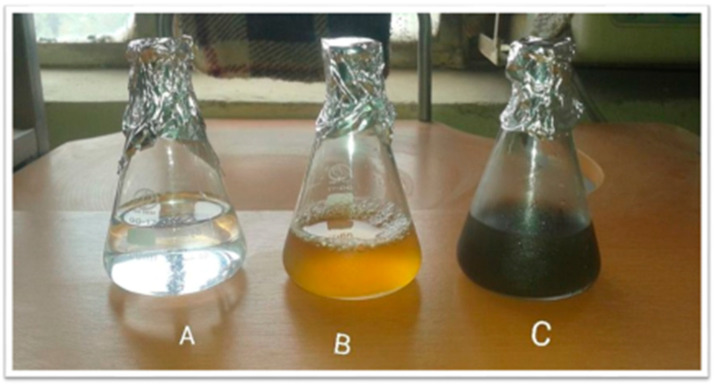
(**A**) Silver nitrate solution, (**B**) plant extract, and (**C**) silver nitrate solution + extract. A color change can be observed from light brown to dark brown. While the precursor silver nitrate solution was colorless (transparent).

The biosynthesized AgNPs have changed from colorless to brown, indicating that Ag^+^ has been reduced to Ag (Figure 1).

### 2.2. Characterization of Biosynthesized Silver Nanoparticles

#### 2.2.1. Analysis of the UV-Visible Spectrum

**Figure 2:**
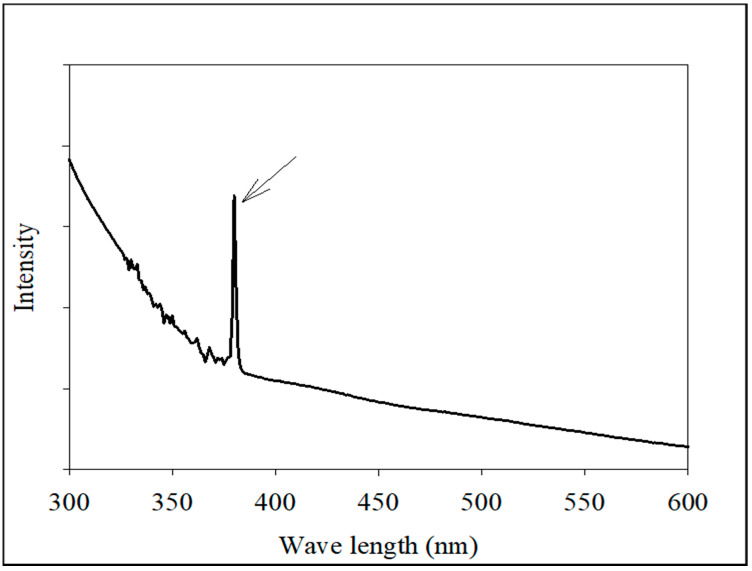
Shows the absorption spectra of Biosynthesized TT-AgNPs and the arrow shows the absorbance peak at 380 nm.

UV-Vis absorption spectroscopy was used to detect the presence of *Tribulus terrestris* AgNPs. TT-AgNPs’ distinctive absorbance peaks were discovered at 380 nm (Figure 2). The finding demonstrated the bio-reductive function of biomolecules present in the extract of *T. terrestris* seeds, which enabled the successful formation of TT-AgNPs.

#### 2.2.2. Analysis of Functional Groups Using FTIR Spectroscopy

**Figure 3:**
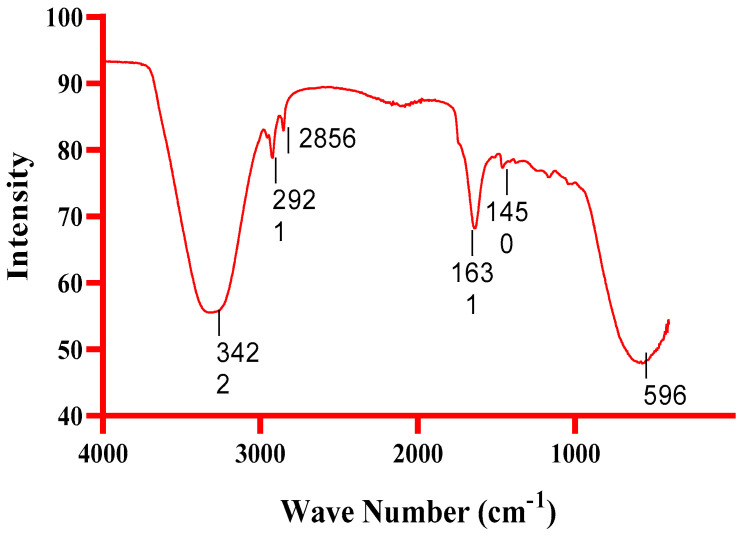
FTIR spectrum of biosynthesized TT-AgNPs that displays the bonds between various capping biomolecules of *Tribulus tristrus*.

Using an FTIR spectrophotometer with a scan range of 400–4000 cm^−1^, the functional groups of TT–AgNPs were determined (Figure 3). NPs transmittance peaks were noticed at 596, 1450, 1631, 2856, 2921, and 3422 cm^−1^ in the FTIR spectra of *Tribulus terristris* seed extract. Functional groups, such as carboxylic acid, ether, ester, phenol, or alcohol, may have caused the O-H-extending vibration peak at 3422 cm^−1^. The phytochemical’s presence of an aromatic component is indicated by the tiny peak at 2921 cm^−1^, which represents the vibration of C-H stretching shown in Table 1. The stretching of the C-H bond in the presence of aromatic aldehydes and CO_2_ is indicated by the band at 2856 cm^−1^. A protein-specific peak at 1631 cm^−1^ was linked to the stretching vibrations of the C=N and C=C bonds. Stretching of the N-H bond indicated the presence of amide linkages in proteins, identified by a faint signal at 1450 cm^−1^. The C-X stretch indicated the presence of alkyl halide, as represented by the peaks at 596 cm^−1^. According to FTIR analysis, *Tribulus tristrus* contained bioactive elements that could reduce and stabilize AgNO_3_NPs. FTIR analysis showed the presence of several *Tribulus tristrus* biomolecules and their role in the biogenic reduction, production, and stabilization of AgNO_3_ NPs.

#### 2.2.3. XRD Analysis

**Figure 4:**
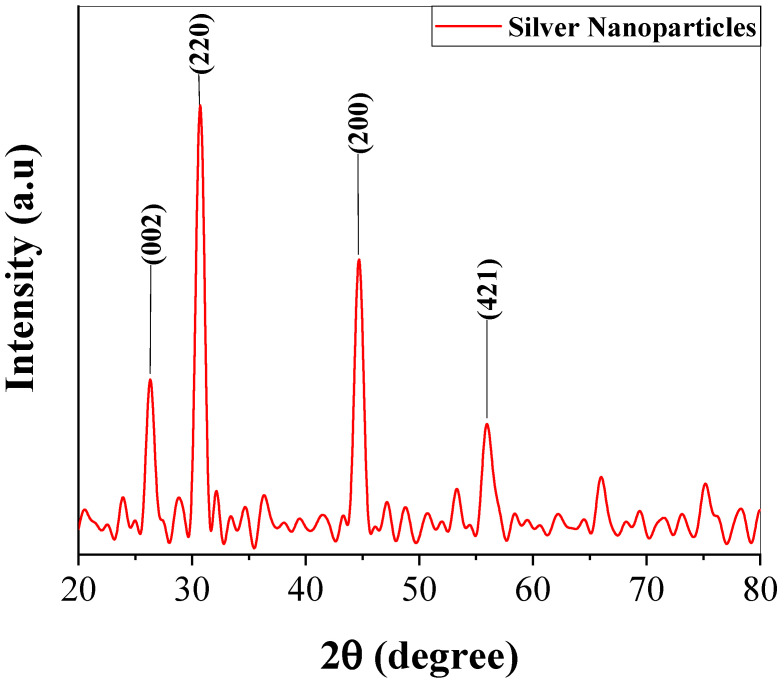
The XRD pattern of biosynthesized TT-AgNPs.

It is evident from the XRD spectrum given in Figure 4 that there were four distinct diffraction peaks in the 2θ range of 20° to 60°. Intense peaks at 26.32°, 30.70°, 44.70°, 56.07°, 53.75°, 66.28°, and 75.32° (corresponding to the lattice plane [25]), respectively found in line with JCPDS-04-0783, indexed the flat surface of the cubic face-centered silver. The result revealed the crystalline structure of biosynthesized TT-AgNPs.

#### 2.2.4. SEM Morphological Analysis of Biosynthesized TT-AgNPs

**Figure 5:**
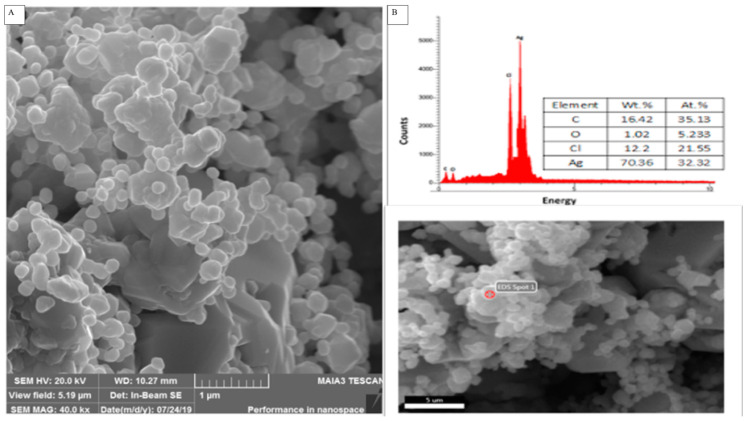
(**A**) SEM analysis of silver nanoparticles produced from *Tribulus terrestris* seed extract; (**B**) EDX of silver nanoparticles.

**Figure 6:**
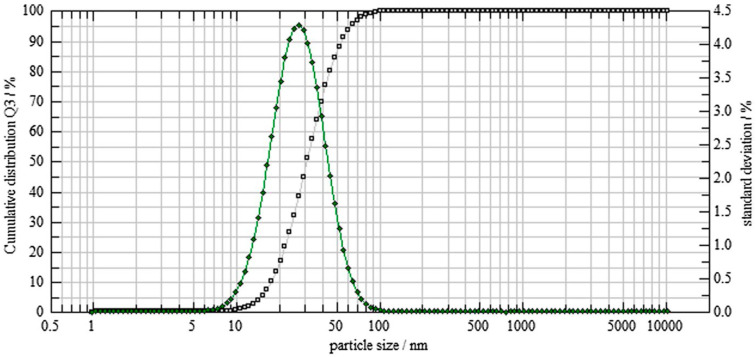
Particle size analysis of AgNPs using the DLS image system.

By using SEM analysis, the structural characteristics of the biosynthesized TT-AgNPs were examined. The synthesized AgNPs appeared as loose clusters and were smooth and spherical, with generally uniform size (Figure 5A). This demonstrates the AgNPs’ stability, spherical shape, and nanoscale dimensions. The elemental analysis of the biosynthesized nanoparticles is revealed by the EDX. The EDX profile in Figure 5B exhibited a strong signal for the presence of Ag atoms; additional peaks were seen for the biomolecules C, O, and Cl that were used to cap the surface of these nanoparticles. The size dispersion of the synthesized TT-AgNPs was assessed using dynamic light scattering (DLS) by a particle size analyzer. The biosynthesized AgNPs were found to be sphere-shaped, and the typical size of the NPs was 22 nm (Figure 6).

### 2.3. In Vitro Anti-Diabetic Assays

#### 2.3.1. Effects of TT-AgNPs on Uptake of Glucose by Yeast Cells in a 5 mM Glucose Solution

**Figure 7:**
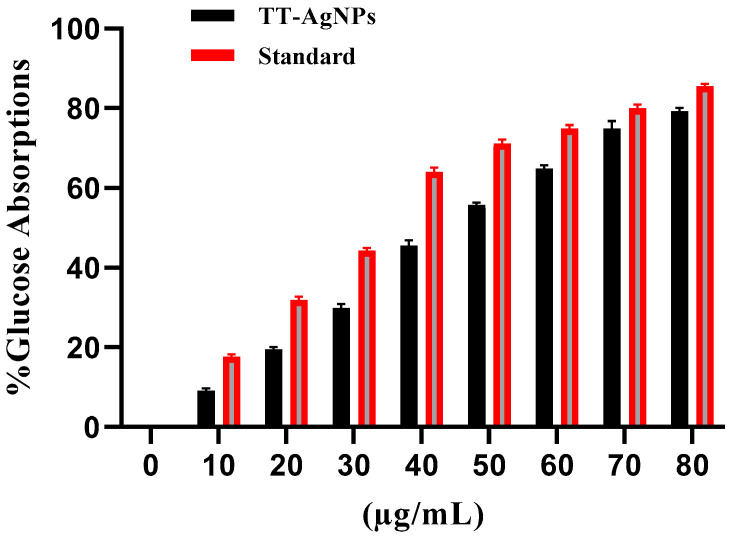
Uptake of glucose by yeast cells using TT-AgNPs.

Using glucose uptake by yeast cell assays, the effects of various concentrations of TT-AgNPs were examined in a 5 mM solution of glucose. The average glucose uptake percentage by yeast cells was 9.35 ± 0.59%, 19.29 ± 0.52%, 30.99 ± 1.06%, 45.61 ± 1.34%, 56.14 ± 0.62%, 65.49 ± 0.83%, 74.85 ± 1.90%, and 78.45 ± 0.84% at concentrations of 10, 20, 30, 40, 50, 60, 70, and 80 (µg/mL) of TT-AgNPs. The study showed a dose-dependent increase in glucose absorption. Metronidazole, as a standard drug, expressed the maximum glucose absorption as 85.2 ± 0.51% at 80 µg/mL (Figure 7).

#### 2.3.2. Effects of TT-AgNPs on Glucose Adsorption

**Figure 8:**
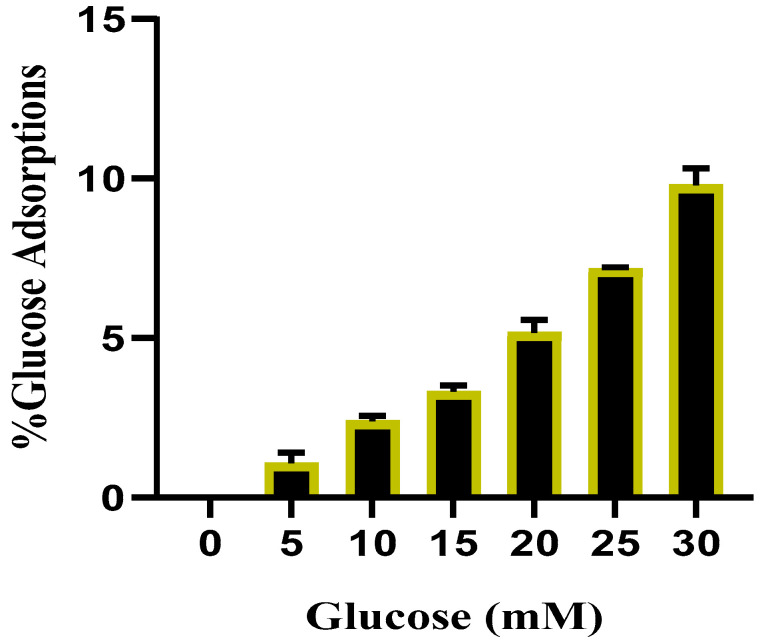
The potential of TT-AgNPs to bind glucose at varying glucose concentrations.

The results of the glucose adsorption assay exhibited that TT-AgNPs have a significant capacity to bind glucose (*p* = 0.05). With rising glucose concentrations (5–30 mM), a consistent increase in glucose adsorption was noticed. At a concentration of 5 mM, the TT-AgNPs glucose adsorption percentage was 1.45 ± 0.31%, and at a concentration of 30 mM, it was 10.40 ± 0.52%. This result showed that the adsorption capacity increased with the increase in glucose. The percentage of TT-AgNP’s glucose adsorption potential at different glucose concentrations is shown in Figure 8.

#### 2.3.3. Effects of TT-AgNPs on Inhibition of α-Amylase Inhibition

**Figure 9:**
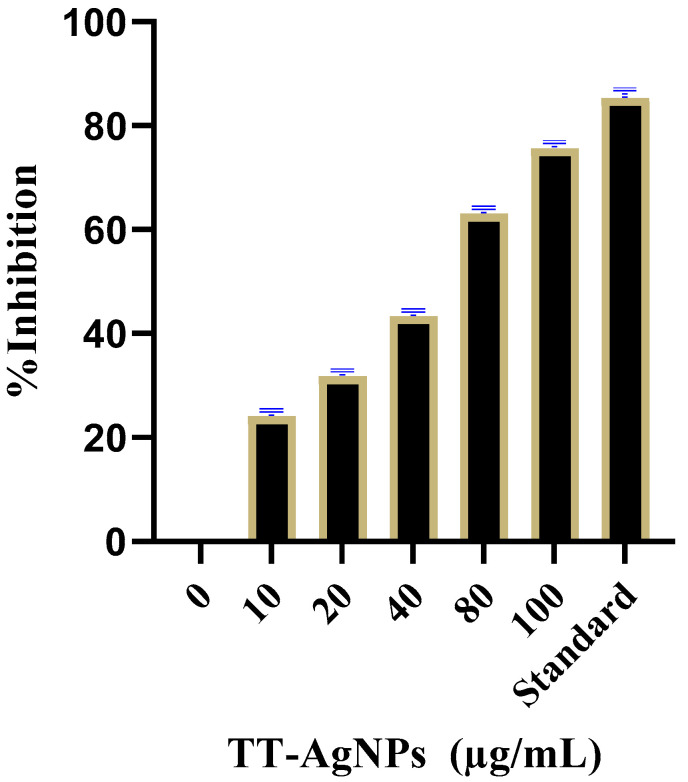
Shows the percentage of the alpha-amylase inhibitory potential of TT-AgNPs at different doses.

The α-amylase inhibitory capacity of TT-AgNPs was evaluated at different doses, i.e., 10, 20, 40, 80, and 100 (µg/mL), and the percent inhibition was 25.1 ± 0.13%, 32.8 ± 0.18%, 42.35 ± 0.22%, 63.15 ± 0.25%, and 75.68 ± 0.11%, respectively; and that of the standard drug acarbose at 100 µg/mL was 85.80 ± 1.52%. The inhibition of α-amylase consistently increased with concentration, proving dose-dependent inhibition (Figure 9).

### 2.4. In Vivo Antidiabetic Assay Results

#### 2.4.1. Assessment of Blood Glucose Level and Body Weight of Experimental Animals Administered with TT-AgNPs

**Figure 10:**
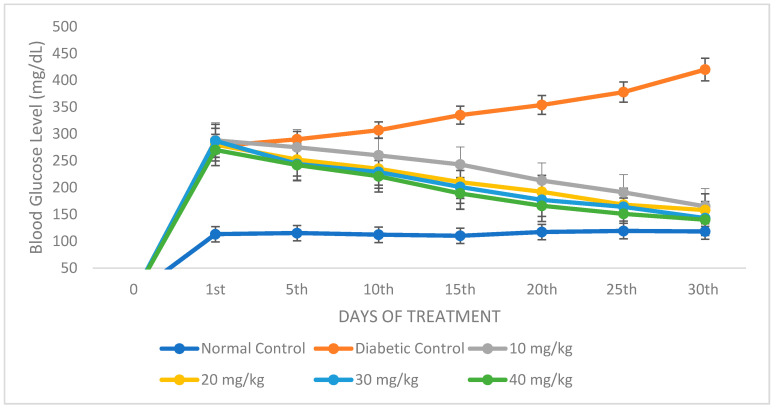
Impacts of TT-AgNPs on blood glucose in streptozotocin-induced diabetic mice and normal controls.

STZ administration led to elevated blood glucose levels and a discernible loss of body weight (Figure 10). The blood glucose level considerably dropped in a dose-dependent manner when diabetic mice were administered 10, 20, 30, or 40 mg/kg of TT-AgNPs for 30 days.

#### 2.4.2. Histopathological Examination of the Liver

**Figure 11:**
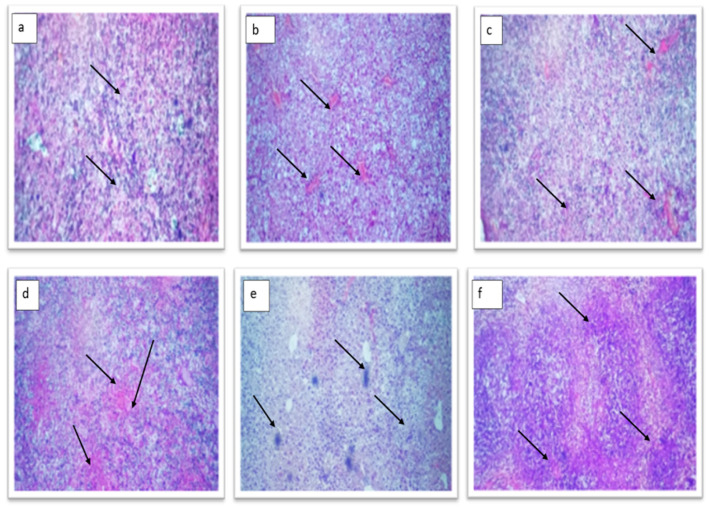
Histopathology of the liver. (**a**) Group-I: normal control group; (**b**) Group-II: diabetic control group; (**c**) Group-III: treated with 10 mg TT-AgNPs; (**d**) Group-IV: treated with 20 mg TT-AgNPs; (**e**) Group-V: treated with 30 mg TT-AgNPs; (**f**) Group-Vi: treated with 40 mg TT-AgNPs.

Experimental animals’ liver tissue was examined under a microscope to show how the TT-AgNPs lessen cellular damage. The diabetic control group (Group-II) displayed the same normal cell architecture as the control group (Group-I), but with obstructed central veins and mild to moderate periportal inflammation (Figure 11b). The hepatocytes in the 10 mg TT-AgNPs-treated group (Group-III) displayed normal architecture and morphology (Figure 11c). The 20 mg TT-AgNPs-treated group (Group-1V) also displayed normal architecture and normal hepatocyte morphology (Figure 11d). The hepatocytes in the group (Group-V) that received 30 mg of TT-AgNPs display normal architecture (Figure 11e). Similarly, the hepatocytes in the 40 mg AgNPs-treated group (Group-V1) showed normal architecture (Figure 11f).

#### 2.4.3. Histopathological Examination of Diabetic Mice Pancreas

**Figure 12:**
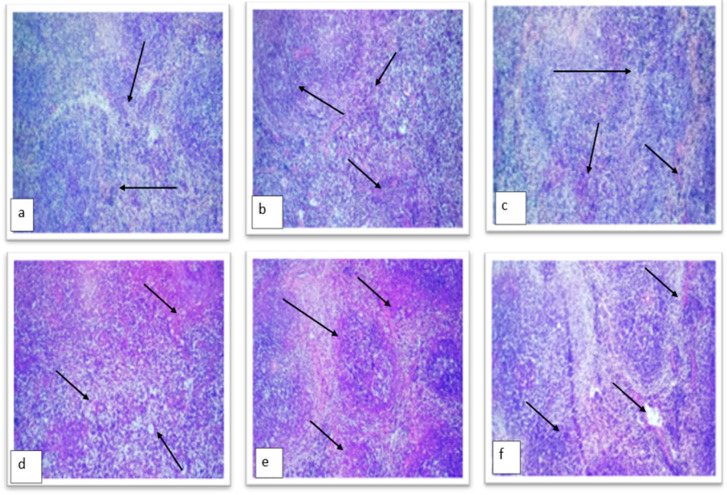
Histopathology of the pancreas. (**a**) Group-I: normal control; (**b**) Group-II: diabetic control; (**c**) Group-III: treated with TT-AgNPs 10 mg; (**d**) Group-IV: treated with TT-AgNPs 20 mg; (**e**) Group-V: treated with TT-AgNPs 30 mg. (**f**) Group-V: treated with TT-AgNPs 40 mg.

Results revealed that the normal control showed an unremarkable pancreas with evenly and densely distributed β cells (Figure 12a). However, some scattered islet cells were observed in groups treated with TT-AgNPs dose-dependently, as depicted in Figure 12b. The untreated diabetic control groups showed highly distorted islet cells and acinar cells in their pancreas. While the β cells in the 10 mg TT-AgNPs treated group (Group-III) displayed normal architecture and morphology (Figure 12c), the 20 mg TT-AgNPs treated group (Group-1V) also displayed normal architecture and normal β cell morphology (Figure 12d). The β cells in the group (Group-V) that received 30 mg of AgNPs display normal architecture (Figure 12e). Similarly, the β cells in the 40 mg AgNPs-treated group (Group-V1) exhibited normal architecture (Figure 12f).

## 3. Discussion

Diabetes mellitus is a serious metabolic illness marked by excessive blood sugar levels and inadequate treatment that causes several chronic complications. The pancreas is the main organ that senses blood glucose levels and regulates insulin accordingly [26]. Researchers are looking for new, yet natural, remedies that could regulate blood glucose levels without causing any side effects. Therapeutic herbs have been found to be better alternatives. Diabetes can be successfully treated with the help of the bioactive substances found in medicinal plants. Additionally, biologically synthesized AgNPs are majorly considered in nanomedicine as they enhance medicinal properties [26].

Because of the widespread issues related to environmental concerns, “green” and environmentally friendly procedures in chemistry and chemical technologies are becoming more and more popular. One of the most widely used nanomaterials is silver, which is produced in five hundred tons annually and is expected to expand in the next few years. Along with its significant contributions to the fields of high-sensitivity biomolecular detection, catalysis, biosensors, and medicine, it has also been recognized for its potent inhibitory and bactericidal effects, as well as its anti-fungal, anti-inflammatory, antidiabetic, anti-cancer, and anti-angiogenic activities [27]. In this study, eco-friendly, bio-mediated silver nanoparticles were synthesized using the methanolic extract of *Tribulus terrestris* seeds. A quick, simple, and entirely green biosynthetic process was used to synthesize silver nanoparticles, using methanolic *T. terrestris* seed extract as the reducing and capping agent. *T. terrestris* seed extract quickly reduced silver ions, producing highly crystalline silver nanoparticles as a result. By analyzing spectra with a UV-Vis spectrophotometer, the formation of the silver nanoparticles was confirmed. The synthesized silver nanoparticles may have been stabilized by interactions between carboxylic groups, carbonyl groups, and the flavonoids found in the *T. terrestris* extract, according to FT-IR (Fourier Transform Infrared) data. The XRD spectrum given in Figure 4 showed that there were four distinct diffraction peaks in the 2θ range of 20° to 60°. Intense peaks at 26.32°, 30.70°, 44.70°, 56.07°, 53.75°, 66.28°, and 75.32°, which indexed the planes of the cubic face-centered silver. By using SEM analysis, the structural characteristics of the biosynthesized TT-AgNPs were examined. The synthesized TT-AgNPs appeared as loose clusters and were smooth and spherical, with a generally uniform size, according to the SEM data. The EDX profile in Figure 5B shows a strong signal for the presence of Ag atoms; additional peaks were seen for the biomolecules C, O, and Cl that were involved in the capping on the surface of these nanoparticles. The result revealed the crystalline structure of green synthesized TT-AgNPs, and similar results were reported in previous studies of biosynthesized AgNPs used for biomedical applications [28,29]. Similarly, plasmonic nanoparticles, which are extremely strong absorbers and scatterers of light, are used in lateral flow diagnostics, surface-enhanced spectroscopy, labeling, and color-changing sensors. By changing nanoparticle size, shape, and composition, the optical response can be tuned from the ultraviolet through the visible to the near-infrared regions of the electromagnetic spectrum. Plasmonic nanoparticles, including gold, silver, and platinum particles, are discrete metallic particles that have unique optical properties due to their size and shape and are increasingly being incorporated into commercial products and technologies. These technologies, which span fields ranging from photovoltaics to biological and chemical sensors, take advantage of the extraordinary efficiency of plasmonic nanoparticles at absorbing and scattering light [30,31].

The yeast cell glucose absorption method has been used to assess the antidiabetic efficacy of numerous natural plants. The process of facilitated diffusion may be involved in transporting glucose into the yeast cell. When most of the glucose is utilized or transformed into other metabolites, the concentration of glucose within the cell drops. This situation promotes the high uptake of glucose into the cell [32]. In this study, a methanolic extract of silver nanoparticles was examined in a 5 mM solution of glucose. In the methanolic extract of silver nanoparticles, the minimum percentage of glucose absorption was measured at low concentrations, while the maximum percentage was observed at high concentrations. Thus, demonstrating that the selected methanolic extract of silver nanoparticles has antidiabetic potential. The present results are in line with [33] on the green biosynthesis of silver nanoparticles from *Tephrosia tinctoria* and their potential antidiabetic activity.

The α-amylase enzyme starts the digestion of carbohydrates present in food; this digestion begins in the mouth and is completed in the small intestine. Since alpha-amylase is a carbohydrate-metabolizing enzyme, inhibiting this enzyme will block carbohydrate breakdown, resulting in decreased postprandial hyperglycemia. Thus far, inhibition of this enzyme has been shown to be a substantial and well-targeted therapeutic strategy for controlling hyperglycemia in type 2 diabetes [32]. In the current investigation, it was found that the TT-AgNPs suppressed alpha-amylase activity by 75.68% at 100 g/mL. The results of this study are supported by those of Riaz et al. [32] regarding the evaluation of a new medication against type 2 diabetes based on a polyherbal extract.

The remaining glucose from digestion may be retained in the intestine, which would prevent it from being absorbed through the adsorption process, from diffusing into the blood, and from increasing the risk of glucotoxicity. High levels of glucose adsorption were revealed by the extracts’ adsorption capacity through glucophage activity. Reduced glucose levels help to lessen the damaging effects of elevated plasma glucose on hemoglobin and other proteins, reducing the absorption of glucose and the development of comorbidities such as retinopathy, neuropathy, and nephropathy in diabetic patients [34]. In the present study, the capacity of glucose adsorption by TT-AgNPs increased with increasing glucose concentration in a dose-dependent manner. The outcome of this study was in accordance with [35] on the potential of some native plants to adsorb glucose.

Streptozotocin is frequently used to induce diabetes mellitus in animals [36]. In this study, blood glucose levels increased after STZ administration. When diabetic mice were treated for 30 days with AgNPs, this condition was reversed, causing a substantial, dose-dependent decrease in blood sugar levels. The blood glucose level at day 30 was dramatically lowered by the AgNPs at a dose of 40 mg/kg. This study is in line with [37] on the anti-diabetic activity potentials of Salacia chinensis stem extract.

From microscopic examination of the pancreas, it was observed that the normal control showed a remarkable pancreas with equally and densely scattered β cells. However, those treated with TT-AgNPs dose-dependently showed sporadic islet cells, as seen in Figure 10. The pancreas of the untreated diabetic control groups exhibited severely deformed islet and acinar cells. Liver histology revealed that the normal control group exhibited normal cell architecture, while the diabetic control group showed normal architecture but a congested central vein. While the TT-AgNPs-treated group showed normal architecture and morphology of hepatocytes. This observation is in accordance with [38] on the evaluation of the antidiabetic activity of biosynthesized silver nanoparticles using Pouteria sapota.

## 4. Methods and Materials

### 4.1. Extract Preparation of TT Seed

*T. terrestris* seeds were purchased from the local market, identified, and specimens placed in the herbarium of the Department of Botany, Abdul Wali Khan University Mardan (AWKUM), with an accession number of Awkum. Bot. 423.1.20. The crude methanolic extract of *T. terrestris* seeds was made by the maceration method according to the procedure of [39]. *T. terrestris* seeds were rinsed under running tap water to clean them, air dried for 2 weeks at 30 °C, and then powdered using a plant grinder (Panasonic Model MX-AC210, Osaka, Japan). Fifty gm of *T. terrestris* seed powder was weighed and added to 600 mL (1:12) of 96% methanol and stored at room temperature (25 ± 3 °C). The mixture was then stirred for 48 h in an orbital shaker (Cole-Parmer Model EW-51700-14, Cole-Parmer, Vernon Hills, IL, USA) at 200 RPM. Using Soxhlet’s apparatus, the extract was then concentrated.

### 4.2. Synthesis of Silver Nanoparticles (AgNPs)

For the biosynthesis of TT-AgNPs, a 100-milliliter solution of silver nitrate (1 mM) and 1 gm of T.T. seed extract were combined. Using a magnetic stirrer hotplate, the reaction mixture was continually heated below the boiling point (70 °C) for 4 h. The color change of the solution indicated that Ag^+^ had been reduced to Ag. Centrifugation was used to separate the resultant AgNPs at 10,000× *g* for 15 min. To eliminate impurities, the pellet was washed three to four times with methanol and deionized water. To reduce silver nitrate’s photoactivation, the reaction was carried out in a dark room. For further use, the precipitated TT-AgNPs were lyophilized, wrapped in aluminum foil, and kept in a cool, dry place.

### 4.3. Characterization of Biosynthesized Silver Nanoparticles

#### 4.3.1. UV-Visible Spectral Analysis

The formation of biogenic TT-AgNPs was confirmed and characterized by the UV-Vis spectrum using a Shimadzu UV-visible spectrophotometer (UV-1800, Kyoto, Japan).

#### 4.3.2. FTIR Spectral Analysis

To identify distinct functional groups in TT-AgNPs, Fourier transformed infrared spectroscopy (FTIR) (IRTracer-100, Shimadzu, Kyoto, Japan) was used.

#### 4.3.3. X-ray Diffraction Technique

The X-ray diffraction method was used to analyze the crystal structure of TT-AgNPs (Model-D8 Advance, Bruker, Bremen, Germany).

#### 4.3.4. Scanning Electron Microscopy (SEM) and Energy-Dispersive X-ray Spectroscopy (EDX) Analysis

A high-resolution image of the surface of the nanoparticles is produced by SEM analysis, which includes details about their size, composition, shape, electrical conductivity, topography, and other characteristics. A SEM (Hitachi, S-4300SE, Tokyo, Japan) was used to assess the morphology and size of TT-AgNPs, and Vega TC software was used for the analysis. Both SEM and energy-dispersive X-ray spectroscopy (EDX) were used to evaluate the morphology of TT-AgNPs and perform chemical composition analysis.

### 4.4. Anti-Diabetic Assays (In Vitro)

The anti-diabetic efficacy of the biosynthesized AgNPs was assessed using three different antidiabetic assays.

#### 4.4.1. Uptake of Glucose by Yeast Cell Assay

This assay was conducted with minor changes to the procedure in [40]. 1 mL of 5 mM glucose solutions were mixed with different concentrations (10 µg/mL to 80 µg/mL) of TT-AgNPs and incubated for 10 min at 37 °C.

The reaction was started by the addition of 10%/V yeast suspension to the mixture. The mixture was vortexed, then incubated for 60 min at 37 °C, followed by a 5-min centrifugation at 3800 rpm. A spectrophotometer (UV 5100B spectrophotometer, Shanghai Metash Instrument Co., Ltd., Shanghai, China) was used to measure the concentration of glucose in the supernatant. The technique was carried out three times.

The glucose uptake by yeast cells was calculated by the following formula:

$$\%\;\mathrm{Glucose}\;\mathrm{uptake}=\frac{\mathrm{Absorbance}\;\mathrm{Control}\;-\;\mathrm{Absorbance}\;\mathrm{Test}\;\mathrm{sample}}{\mathrm{Absorbance}\;\mathrm{Control}}\times 100$$

#### 4.4.2. Glucose Adsorption Assay

The potential of TT-AgNPs to adsorb glucose was assessed by the glucose adsorption technique. This assay was performed following the method of [41] to measure the amount of glucose bound. 1 g of the TT-AgNPs was mixed with a 100-milliliter solution of glucose (5–30 mM glucose). The resultant mixture was shaken at room temperature for up to 6 h, then centrifuged at 4800 rpm for 20 min.

The following formula was used to determine the amount of glucose t in the supernatant:

Bound Glucose = G1 − G6 × sample volume

Sample’s weight:

G1—concentration of glucose before reaction;

G6—concentration of glucose after 6 h.

#### 4.4.3. α-Amylase Inhibition Assay

To produce glucose and maltose, the enzyme alpha-amylase hydrolyzes the alpha bonds present in polysaccharides. As it raises blood glucose levels, its suppression will result in a fall in blood glucose levels. Following the method of [42], porcine pancreatic amylase (500 µL) in phosphate buffer, standard drug (250 µL), and methanolic AgNPs reaction mixtures were made and incubated for 20 min at 37 °C. Followed by the addition of 250 µL of PBS (100 mM) and 1% starch to the reaction mixture and incubation for 60 min at 37 °C. After adding 1 mL of the di-nitro-salicylate color reagent and heating for 10 min, the absorbance was determined at 540 nm. Using the following formula, % inhibition was calculated.

$$\%\;\mathrm{Inhibition}=\frac{\mathrm{Control}\;\mathrm{Absorbance}\;-\;\mathrm{sample}\;\mathrm{Absorbance}}{\mathrm{Absorbance}\;\mathrm{Control}}\times 100$$

### 4.5. In Vivo Anti-Diabetic Activity

#### 4.5.1. Experimental Animals and Conditions

In the current study, healthy adult BALB/C mice (25–30 g, 5 weeks old) were purchased from the Veterinary Research Institute Peshawar. The animals were kept under regulated, appropriate conditions (22±2 °C, 60% relative humidity, 12 h of light and dark cycles), provided standard chow, and given free access to water. All studies were conducted strictly in accordance with the National Research Council’s rules on the care and use of laboratory animals, and the experimental protocol was approved by the Experimentation Ethical Committee of the Department of Zoology, Abdul Wali Khan University, Mardan, Pakistan.

#### 4.5.2. Inducing Diabetes in Mice

Freshly prepared streptozotocin (STZ) in Na-citrate buffer (0.1 M), pH 4.5, and 50 mg/kg body weight were administered intraperitoneally to the mice to induce diabetes. Whereas citrate buffer alone was administered to the control group. The induction of diabetes was confirmed by a blood glucose test after 72 h of streptozotocin injection.

#### 4.5.3. Experimental Design

The animals were divided into six study groups randomly (*n* = 5). Five groups of successful diabetic-induced mice were divided as follows:

Group I: Normal Control (0.5 mL of 0.1 M citrate buffer, pH 4.5);

Group II: Diabetic Control (STZ 50 mg/kg);

Group III: Diabetic mice treated with AgNPs (10 mg/kg);

Group IV: Diabetic mice treated with AgNPs (20 mg/kg);

Group V: Diabetic mice treated with AgNPs (30 mg/kg);

Group VI: Diabetic mice treated with AgNPs (40 mg/kg).

#### 4.5.4. Assessment of Body Weight and Blood Sugar Level

The weight of the mice was continuously checked at intervals of five days. Periodically, on every 5th day, the fasting glucose level was checked using the “rupturing tail vein method” by a one-touch glucometer (LifeScan, Inc., Milpitas, CA, USA) for one month.

## 5. Conclusions

There are many medications available to cure diabetes, but they have many adverse effects. To minimize these adverse effects, new medications must be introduced. In the current work, an effort has been made to assess the outcomes of several in vitro and in vivo assays to confirm the anti-diabetic capabilities of biosynthesized silver nanoparticles. The potential of biosynthesized silver nanoparticles for glucose uptake by yeast cells was evaluated. According to the glucose adsorption test, synthesized silver nanoparticles have a high capacity to bind glucose. Using various doses, the biosynthesized silver nanoparticle’s ability to inhibit amylase was assessed. A dose-dependent inhibition of α-amylase was observed. To the best of our knowledge, this was the first in vitro and in vivo antidiabetic study using the green biosynthesis of silver nanoparticles using plant seeds of *Tribulus terrestris*.

## Figures and Tables

**Table 1:** Characteristic peak data showing the presence of functional groups on the surface of green synthesized silver nanoparticles.

Wave Number (cm^−1^)	Functional Group
3422	O-H stretching
2921	C-H stretching of aromatic compounds
2856	C-H stretching of aromatic compounds
1631	C-N and C-C stretching indicate the presence of proteins
1450	N-H stretch showing amide linkage of proteins
596	C-X stretch indicated the presence of an alkyl halide

## Data Availability

Not applicable.
